# PVCbase: an integrated web resource for the PVC bacterial proteomes

**DOI:** 10.1093/database/bay042

**Published:** 2018-04-24

**Authors:** Nicola Bordin, Juan Carlos González-Sánchez, Damien P Devos

**Affiliations:** 1 Centro Andaluz de Biología del Desarrollo, CSIC, Universidad Pablo de Olavide, Carretera de Utrera, Km. 1, Seville 41013, Spain; 2 CellNetworks, BioQuant, University of Heidelberg, Im Neuenheimer Feld 267, 69120 Heidelberg, Germany; 3 Biochemie Zentrum Heidelberg (BZH), Heidelberg University, Im Neuenheimer Feld 328, 69120 Heidelberg, Germany

## Abstract

Interest in the *Planctomycetes-Verrucomicrobia-Chlamydiae* (PVC) bacterial superphylum is growing within the microbiology community. These organisms do not have a specialized web resource that gathers *in silico* predictions in an integrated fashion. Hence, we are providing the PVC community with PVCbase, a specialized web resource that gathers *in silico* predictions in an integrated fashion. PVCbase integrates protein function annotations obtained through sequence analysis and tertiary structure prediction for 39 representative PVC proteomes (PVCdb), a protein feature visualizer (Foundation) and a custom BLAST webserver (PVCBlast) that allows to retrieve the annotation of a hit directly from the DataTables. We display results from various predictors, encompassing most functional aspects, allowing users to have a more comprehensive overview of protein identities. Additionally, we illustrate how the application of PVCdb can be used to address biological questions from raw data.

**Database URL**: PVCbase is freely accessible at www.pvcbacteria.org/pvcbase

## Introduction 

The *Planctomycetes-Verrucomicrobia-Chlamydiae* (PVC) bacterial superphylum is composed of the three name-giving phyla and some additional ones, like *Lentisphaerae*, ‘*Candidatus* Omnitrophica’ and *Kirimatiellaeota* (1). Despite this diversity, it is now accepted that they form a monophyletic group (2). This bacterial superphylum draws interest because some species display characteristics not frequently observed in bacteria. Examples of these are condensed DNA (nucleoids), extensive inner membrane organization (3), the ability to internalize external compounds before degradation (4), the presence of membrane coat-like proteins linked to the extensive membrane organization (5, 6) and that they were thought to lack peptidoglycan until recently (7, 8). Interesting characteristics relevant for wastewater treatment are shown by some planctomycetes having an anammoxosome, possibly the first true prokaryotic organelle, which allows the bacteria to degrade ammonium anaerobically (9). The exceptional diversity of cell plans displayed by some of the phyla, showcased by *Gemmata obscuriglobus* and some verrucomicrobia (10), has been the subject of controversial interpretations (11, 12).

**Figure 1.**
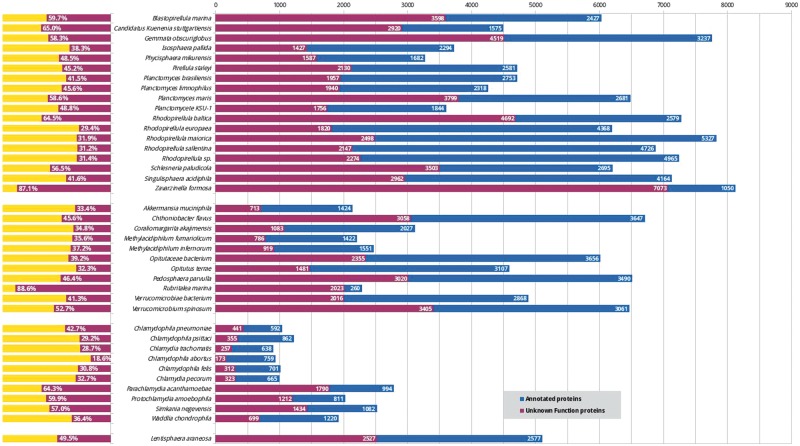
Status of functional annotation of PVC proteins. Total numbers (right) and percentage of total (left) proteins annotated (blue or yellow bars) and unannotated (purple) in each proteomes.

Despite the considerable interest in these bacteria in many fields, including cell biology, evolution (13) and biotechnology (14), these organisms lack a centralized resource for their analysis. While new PVCs are being sequenced, the mean percentage of unannotated proteins constitutes approximately 46% (Figure 1). This issue does not exclusively affect the PVC bacteria since complete genome sequencing rarely translates into a complete characterization of the organism (in UniProt 31% of non-PVC proteomes are uncharacterized) (15). Protein function is not limited to a single feature or description; therefore we developed a pipeline for the simultaneous consideration of many different sequence descriptors. Since our aim is to provide these results to the community of PVC experimentalists, the results were collected in a resource built taking user-friendliness into consideration. Users are able to easily query the results of all functional predictors and download the predictions in bulk for large-scale interrogations.

## Methods

Unless specified otherwise, all tools were used with default parameters.

## Proteomes collection

The proteomes of 39 PVC representative species (17 planctomycetes, 11 verrucomicrobia, 10 chlamydia and 1 lentisphaerae) comprising a total of 173 664 protein sequences, were obtained from the UniProtKB and the NCBI-protein databases. The complete list of PVC species is given in supplementary (See online supplementary material for Table S1).

## Homology-based inference

For every sequence, an homology search was performed using PSI-BLAST (16) with three iterations and default parameters, against the UniProtKB/Swiss-Prot database (release 2015_02) (17). The raw output was parsed to extract all the hits showing an *E*-value below 1E-3 and a minimum coverage of 75% of the query sequence. The first of these matches was selected as the best match and information regarding function under the form of GO description (18), keywords and enzymatic activity was assigned to the query protein. From the remaining matches, GO terms were extracted and counted, and they were reported only if they appeared in at least 10% of the hits.

## Domain analysis

The tool InterProScan (v5.16-55.0) (19) was used to search for protein signatures by scanning their sequences against all its member databases: Pfam (release 28.0), TIGRFAM (release 15.0), PANTHER, ProSite (release 20.113), HAMAP (release 2015_11), PIRSF (release 3.01), Gene3D (release 3.5.0), SUPERFAMILY (release 1.75), PRINTS (release 42.0), SMART (release 6.2) and InterPro (20), using the parameters *-goterms* and *-pa.* Thanks to these options, entries from the InterPro database also provided functional information in the form of terms from GO and KEGG-pathway entries (21).

## Tertiary structure prediction

A series of programs and utilities included in the HHsuite package (v2.0.16) were used. For each sequence, first HHblits (22) was used to construct a high-quality multiple sequence alignment (MSA) by comparing it against the UniProt20 database of template Hidden Markov models (HMMs) (release 2013_03), with the option *-addss* which adds secondary structure information predicted with PSIPRED v3.5 (23) to the resulting MSA. It was then converted to a HMM (.hhm format) with the *hhmake* function. Finally, HHsearch (24) was used to compare it against the HMM template database pdb70 (release 16May15), which is based on the protein data bank (PDB) (25). Every tool was run with default parameters. The results for both comparisons, against UniProt20 and pdb70, were parsed with an *E*-value cut-off of 1E-3. For the latter, functional information in the form of GO terms and EC codes was gathered from SIFTS mapping (26).

## Prediction of signal peptides and transmembrane helices

Signal peptides were predicted with SignalP4.1 (27) using the *gram-* option. Transmembrane helices (TMHs) were predicted with TMHMM (28). The content (%) of integral membrane or transmembrane proteins of the proteome was defined as the fraction of proteins for which at least one TMH was predicted (transmembrane proteins/total number of proteins*100).

## Prediction of protein intrinsic disorder

The IUPred tool (29) was used to predict intrinsically disordered regions and globular domains. The default threshold of 0.5 was used to determine whether a residue was considered as structured or disordered. Three metrics were computed to describe disorder within the proteome (30): (i) the disorder content (%) which was calculated as the fraction of disordered residues in the proteome (total predicted disordered residues/total number of residues*100); (ii) the content (%) of long disordered regions (LDRs), which are defined as those regions where at least 30 disordered residues are predicted continuously along the sequence, calculated as the fraction of residues in those LDRs, (residues in LDRs/total number of residues*100); and (iii) the fraction (%) of highly disordered proteins (HDPs) which are defined as those with >50% of predicted disordered residues in their sequences (number of HDP/total number of proteins*100).

## PVCbase

**Figure 2.**
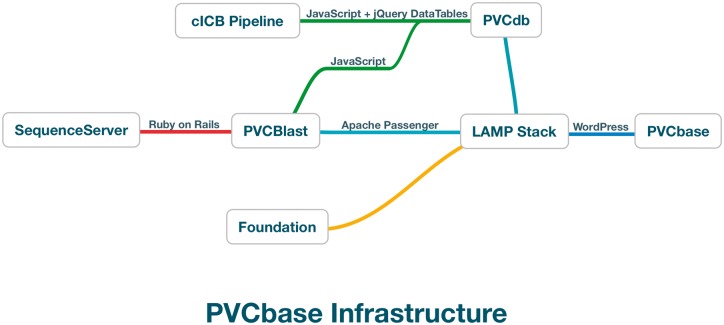
PVCbase organization and relationship between services.

PVCbase is a webserver developed to distribute the predictors results and statistics on disorder and TMHs distributions for the PVC superphylum. The resource acts as a gateway to PVCdb, the BLAST webserver and our secondary structure descriptor, Foundation. PVCbase is built on top of a Linux-Apache-MySQL-Python stack with WordPress as content management system (Figure 2).

## PVCdb

**Figure 3.**
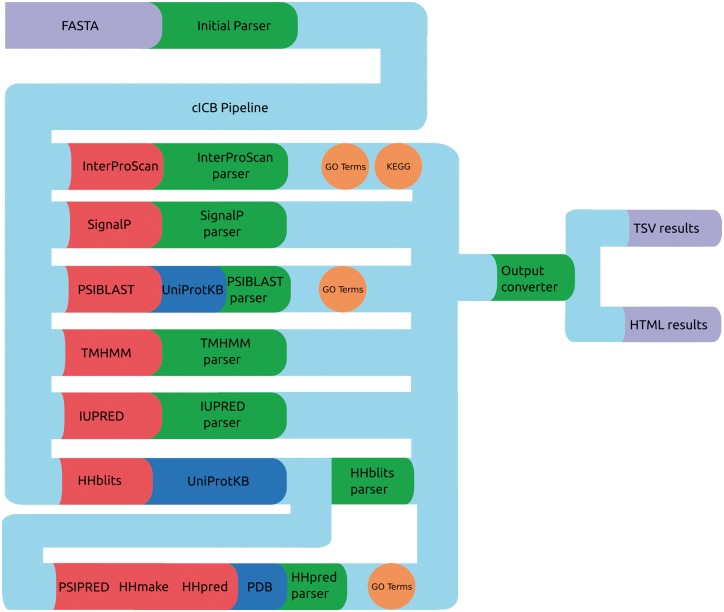
cICB pipeline. Files (purple), Tools (red), databases (blue) and scripts (green) used to generate the annotations for PVCdb. Color spheres indicates additional information obtained through the predictors.

PVCdb data include sequence and structure-based features that were computed using a Python-Perl pipeline that runs, parse and organize the predictors results (Figure 3), generating tabular and HTML web pages for each proteome. The HTML pages include Javascript code that retrieves the query originated from PVCBlast from the URI and pre-filters the jQuery DataTable on load. The standard DataTables plugin was modified in order to allow fixed headers, table prefiltering and paging.

## PVCBlast

PVCBlast is based on the SequenceServer (31) Ruby package running on a RubyOnRails-Passenger-Apache stack. The Ruby was customized to highlight hit significance, links to PVCbase, a sample FASTA file, further sequence checks and a link-out system that connects a hit on a PVC proteome to its annotation in PVCdb. We included some additional controls to the original SequenceServer, such as refresh and back-to-top buttons.

## Foundation

For ease of visualization, we provide a protein features visualization tool, Foundation, that combines secondary structure (predicted by PSI-Pred), Transmembrane helices (by TMHMM) and disorder (by IUPred) predictions. In addition to a quick visualization of sequence feature, an illustration is provided in post-script, allowing the addition of more annotations, such as domains or mutated residues, as well as merging of various images, with limited understanding of postscript scripting. Foundation allows downloading of the raw results from the predictors for further analysis.

## Results

### PVC proteomes

We gathered the proteomes of 39 PVC species comprising 17 planctomycetes, 11 verrucomicrobia, 10 chlamydia and 1 lentisphaerae (See online supplementary material for Table S1). The three main phyla, *Planctomycetes, Verrucomicrobia* and *Chlamydia*, show an important variance of their proteome sizes. As reference, some of the model bacteria like *Escherichia coli* or *Bacillus subtilis* have 4305 and 4197 different proteins, respectively. Chlamydia has very reduced genomes with low protein numbers (mean/median: 1532–1125 proteins). *Chlamydia trachomatis* possesses one of the tiniest proteome, with only 895 proteins. The *Verrucomicrobia* appears to be intermediary (4307/4588) with some close to the size of reduced chlamydial pathogens with around 2000 proteins. In contrast, the *Planctomycetes* displays much larger proteome sizes (5881/6193), which rank them among the bacteria with the biggest genomes and most protein-coding genes. The largest PVC proteomes belong to the *Planctomycetaceae* family, with *Zavarzinella formosa*, *Rhodopirellula maiorica SM1* and *G.obscuriglobus* encoding 8123, 7825 and 7756 proteins, respectively, almost one order of magnitude bigger than the smallest chlamydial proteome, *C.trachomatis* and bigger than some eukaryotes, like the baker’s yeast *Saccharomyces cerevisiae* that encodes 6721 proteins. The biggest planctomycetal genome, *Fimbriiglobus ruber*, has recently been reported to have a size of 12.364 Mbp and it encodes more than 10 000 proteins (32).

For comparison, one of the biggest bacterial genomes is found in *Ktedonobacter racemifer* in the phylum *Chloroflexi*, with 13.7 Mbp and coding for 11 000 proteins (33).

## Usage of PVCbase

### PVCdb

**Figure 4.**
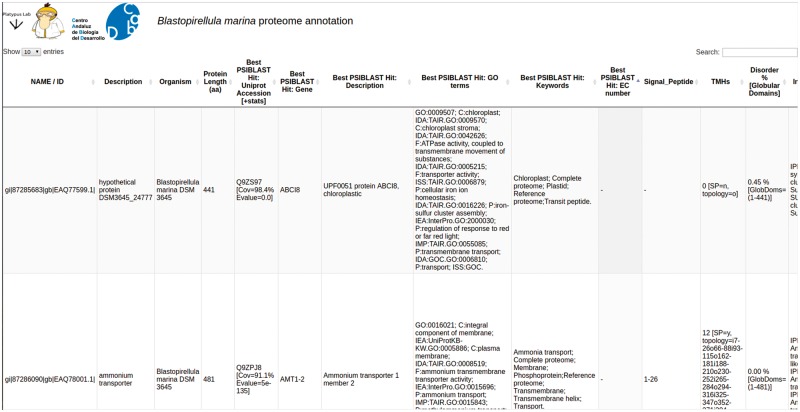
PVCdb. Example of Web interface of PVCdb**.** DataTable of Blastopirellula marina. The figure shows portion of the bacterial annotation. Each column can be sorted and browsed using the built-in search bar.

PVCdb collects protein functional annotations of 39 PVC proteomes. Each proteome can be downloaded as multiFASTA and the corresponding annotation can be either downloaded as a tabular file or easily browsed online. PVCdbs can show a variable amount of entries, based on user choice. Table rows can be sorted by length or alphabetically, while the search bar filters the table and shows only hits that contain the searched keyword. This is helpful to extract subsets of proteins based on localization, process or related to specific activities (Figure 4).

### PVCBlast

**Figure 5.**
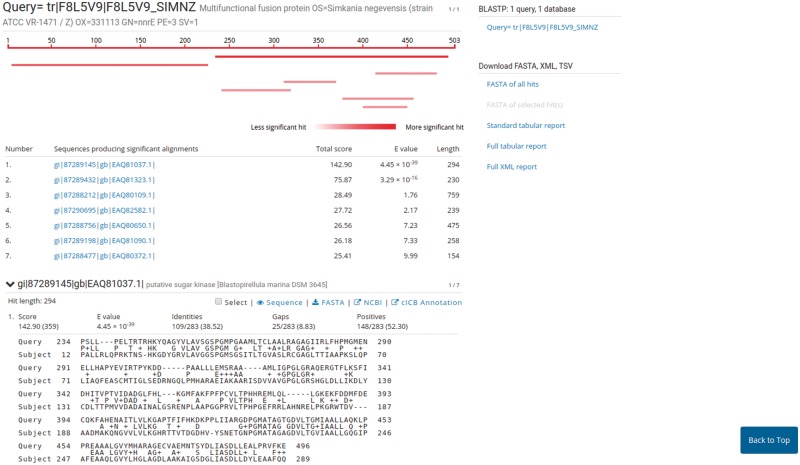
Results page of PVCBlast. The results page of PVCBlast provides the user with the alignments and the ability to download the results in various formats (top right). Among the linkout systems (center), clicking on ‘cICB annotation’ opens the correspondent annotation on PVCdb.

PVCBlast allows the user to perform BLAST searches on the PVC proteomes and genomes. The search box supports drag-and-drop and multiple sequences at once. The BLAST search parameters, such as evalues cutoff and number of alignments, can be customized using the ‘advanced parameters’ bar at the bottom of the page. The results page shows the alignments produced by BLAST, alongside several options for downloading the hits sequences and reports from the tool. The default SequenceServer interface provides a link-out service to NCBI Genbank or UniProt, according to the proteome source. We modified the aligned hits window to indicate the significance of a hit using shades of red. A link-out generator was created to link a hit on a PVC proteome to the corresponding annotation in PVCdb, pre-filtering the DataTable (Figure 5).

### Foundation

**Figure 6.**
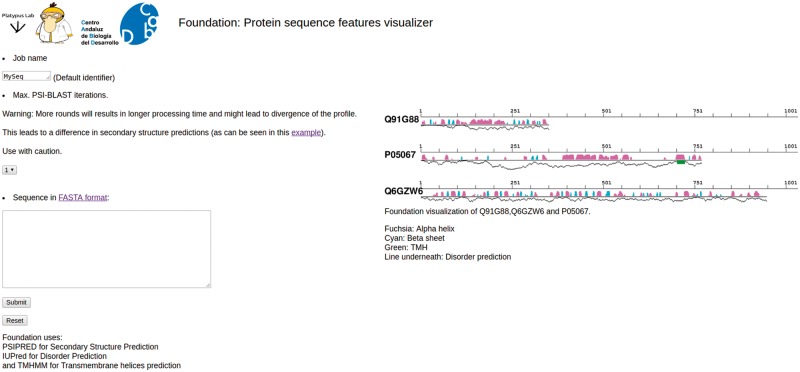
Foundation. Snapshot of Foundation’s main page. The input box allows only single protein sequences. The number of PSIBLAST iterations can be modified using the drop-down menu (center-left). On right side of the page there are some examples of the output and the legend.

Foundation is a tool to quickly visualize the linear and secondary structure features for a provided protein with its secondary structure features. Results can be downloaded as a png or postscript file, as a compressed tar file containing the raw output of the predictors, or can be visualized as an interactive zooming map. Secondary structure features are depicted with different color bars, fuchsia for α helices and cyan for β sheets. TMHs are shown as green boxes and the line underneath the secondary structure shows the disorder level for each amino acid (Figure 6).

## Transmembrane proteins, intrinsic disorder and internal membrane: applications of PVCdb

In order to illustrate one of the possible discoveries made possible by PVCbase that would have been difficult to realize with other currently existing databases, we provide the following example. Planctomycetes cells present internal organizations characterized by the presence of developed membrane organizations which are atypical for bacteria (12). These have been extensively studied in *G.obscuriglobus* by three-dimensional tomography reconstructions that reported an extensive network of internal membranes (3, 6). A separate compartment has also been described in anammox planctomycetes (34). Variations of this cellular structure are believed to be shared by other planctomycetes and verrucomicrobia but are still mostly unexplored (10, 12, 35). We investigated a possible relationship between the presence of internal membrane and the number of transmembrane proteins. We calculated the fraction of proteins with TMHs for each PVC proteome and for each proteome of three reference sets composed of non-PVC bacterial, archaeal and eukaryotic species (see Methods). These reference proteomes are representative of the three domains of life with diverse cellular plans (See online supplementary material for Table S2).

**Figure 7.**
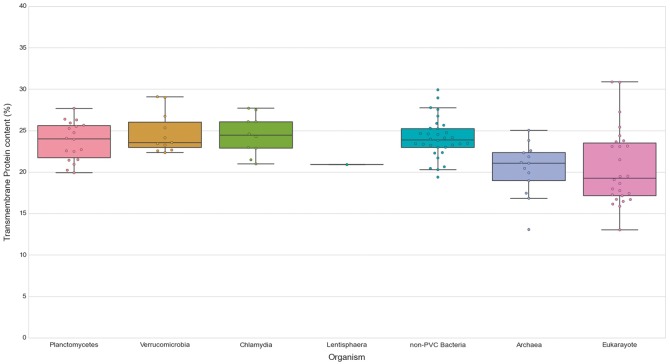
Transmembrane proteins in PVC and representative species from the tree of life. The numbers of TMHs containing proteins, expressed as percentages of the proteomes. Box plots reflect the distribution of the data. The box encloses the quartiles of the dataset, while the whiskers extend to the limits of the distributions. Outliers are determined based on the interquartile range and are not included in the boxes. The middle horizontal line in the box marks the median of the distribution.

We first noticed that bacteria, in general, show a slightly higher content of TMHs than the analyzed species of archaea (*P*-value = 5.00E-04) and Eukaryotes (*P*-value = 6.92E-04). The content in transmembrane proteins showed however no statistical difference for any of the PVC bacterial groups when compared against all other groups and assessed by the Wilcoxon rank-sum test (See online supplementary material for Table S5, Figure 7). Therefore, the PVC genomes possess a smaller fraction of transmembrane protein in comparison to other bacteria (all means and medians between 23 and 24%). Additionally, we compared the fraction of transmembrane proteins to their number of TMHs (See online supplementary material for Table S3). The results provided further evidence for the previous observation. Thus, transmembrane protein content does not seem to be correlated with membrane complexity. This is illustrated by comparing the human and *C.trachomatis* proteomes, which respectively contain 17.41% and 24.58% of proteins containing at least one TMH.

**Figure 8.**
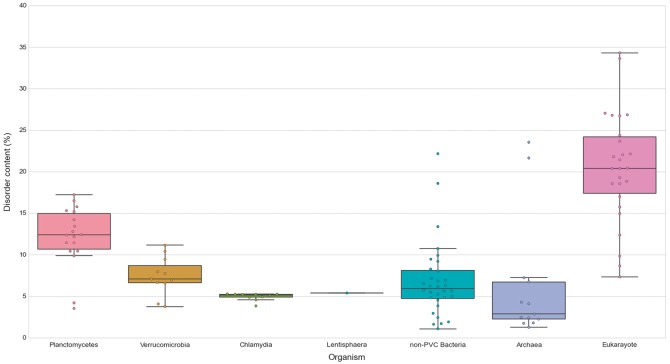
Disorder content in PVC and representative species from the tree of life. The numbers of disordered proteins, expressed as percentages of the proteomes. Box plots reflect the distribution of the data. The box encloses the quartiles of the dataset while the whiskers extend to the limits of the distributions. Outliers are determined based on the interquartile range and are not included in the boxes. The middle horizontal line in the box marks the median of the distribution.

Similarly, we explored the differences in protein structural disorder between these groups. We computed three metrics to describe the intrinsic disorder of the proteomes: the total disorder content, the fraction of residues in LDRs and the fraction of highly disordered proteins (HDPs) (see Methods) (Figure 8; See online supplementary material for Table S4 . As demonstrated elsewhere, disorder content is, for both prokaryotes and eukaryotes, generally independent of the proteome size (36). This observation is also reflected in our data. It is worth noting that the two outliers in the data belonging to archaea, and the largest one from the non-PVC bacteria values, correspond to three extreme halophilic organisms (the archaea *Halobacterium salinarum NRC1* and *Nanosalina sp.*, and the bacterium *Salinibacter ruber*) (See online supplementary material for Figures S1 and S2). This observation agrees with the suggestion that intrinsically disordered proteins may help these organisms adapt to the extreme environments they inhabit. Disordered regions have an increased tolerance against mutations which allows for a higher evolutionary rate that results in extraordinary adaptability (37).

We first observed, as previously reported, that eukaryotes have more disordered proteomes (10), which has been related to the importance of disorder for cellular complexity. We then detected that most planctomycetes show a higher content of disordered proteins (mean/median of 12.15–12.39%) than non-PVC bacteria (6.82–5.95%). This trend is not observed in verrucomicrobia (7.43–7.08%) or chlamydia (4.95–5.14%). A statistical evaluation confirmed both observations: the disorder contents of planctomycetes species are significantly superior to the other three bacterial groups (Wilcoxon rank-sum test *P*-values: P vs V = 7.49E-04, P vs C = 6.64E-04, P vs non-PVC = 2.97E-04) and that the values from the other groups are not statistically different from each other (See online supplementary material for Table S5).

Low complexity, or intrinsically disordered proteins, ise mostly associated with signal transduction, cell-cycle regulation and transcription (38). However, it has been suggested that structural disorder plays a fundamental role in vesicle trafficking pathways (39) and it has been demonstrated that certain unstructured protein domains are highly efficient drivers of membrane curvature. Disordered fragments have a role in membrane coat assembly and vesicle communication, in what has been called the fly-casting mechanism (40). This is especially the case in the clathrin-coated vesicle system, which mediates endocytosis and the early secretory pathway (35). Thus our observation suggests that the significantly higher ratio of disordered proteins in *Planctomycetes* appears to be correlated with the development of their membranes.

## Conclusions

PVCbase offers a convenient one-stop platform for the PVC bacteria community. Its scalability and variety of annotations have already been used in PVC-related publications (41) and newly sequenced organisms will be added regularly. Bulk data collecting also allows users to infer biological discoveries by comparing annotations at the proteome level.

## Availability

PVCbase is freely accessible at http://pvcbacteria.org/pvcbase.

## Supplementary Material

Supplementary Data
