# Supplementary figures and images for: PVCbase: an integrated web resource for the PVC bacterial proteomes
Source: Database (Oxford). 2018 Apr 24;2018:bay042. doi: 10.1093/database/bay042 (PMC5915940; doi:10.1093/database/bay042)

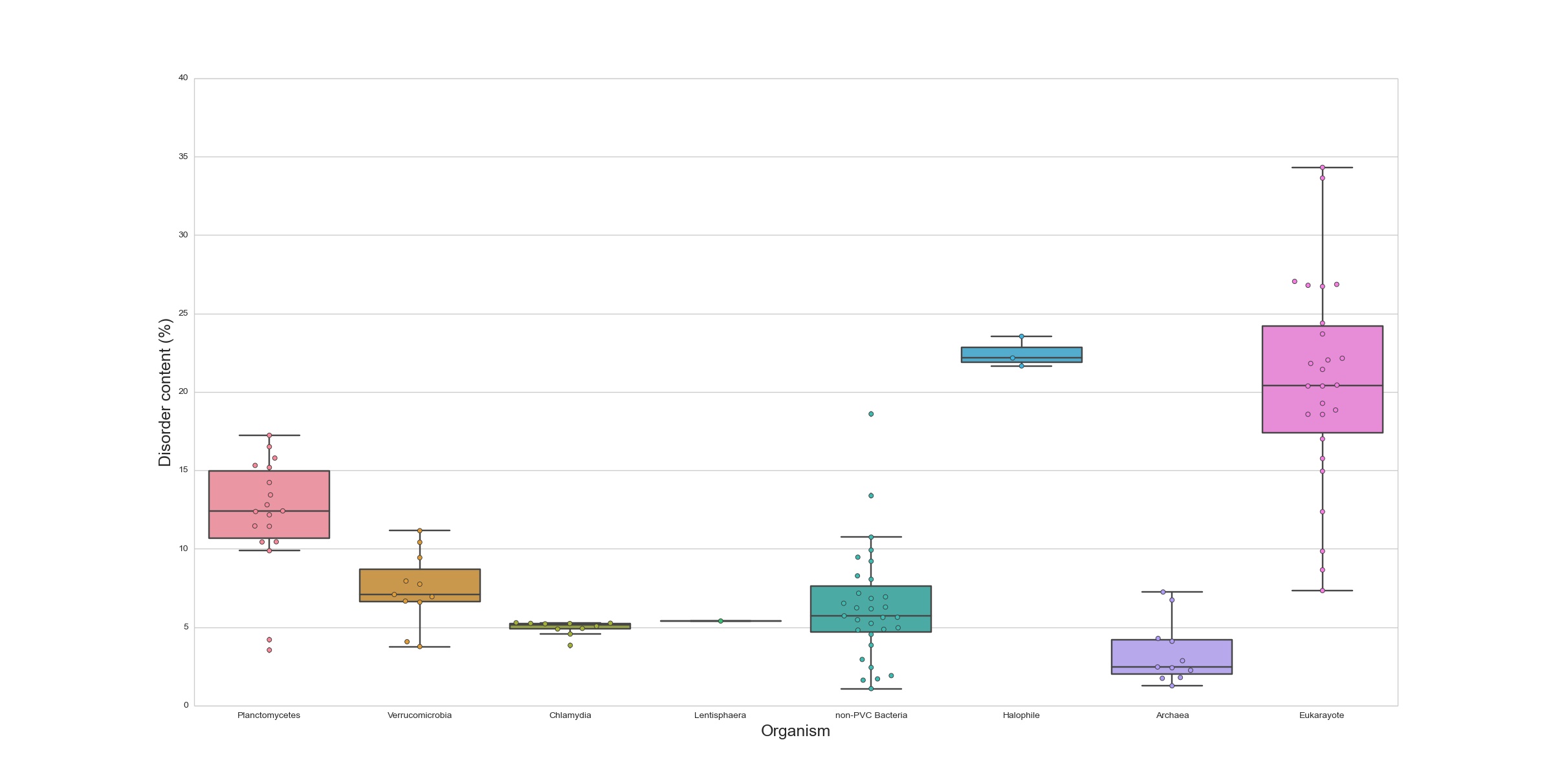

Supplement: Supplementary Data [file bay042_supp.zip › Figure_S1.JPEG]

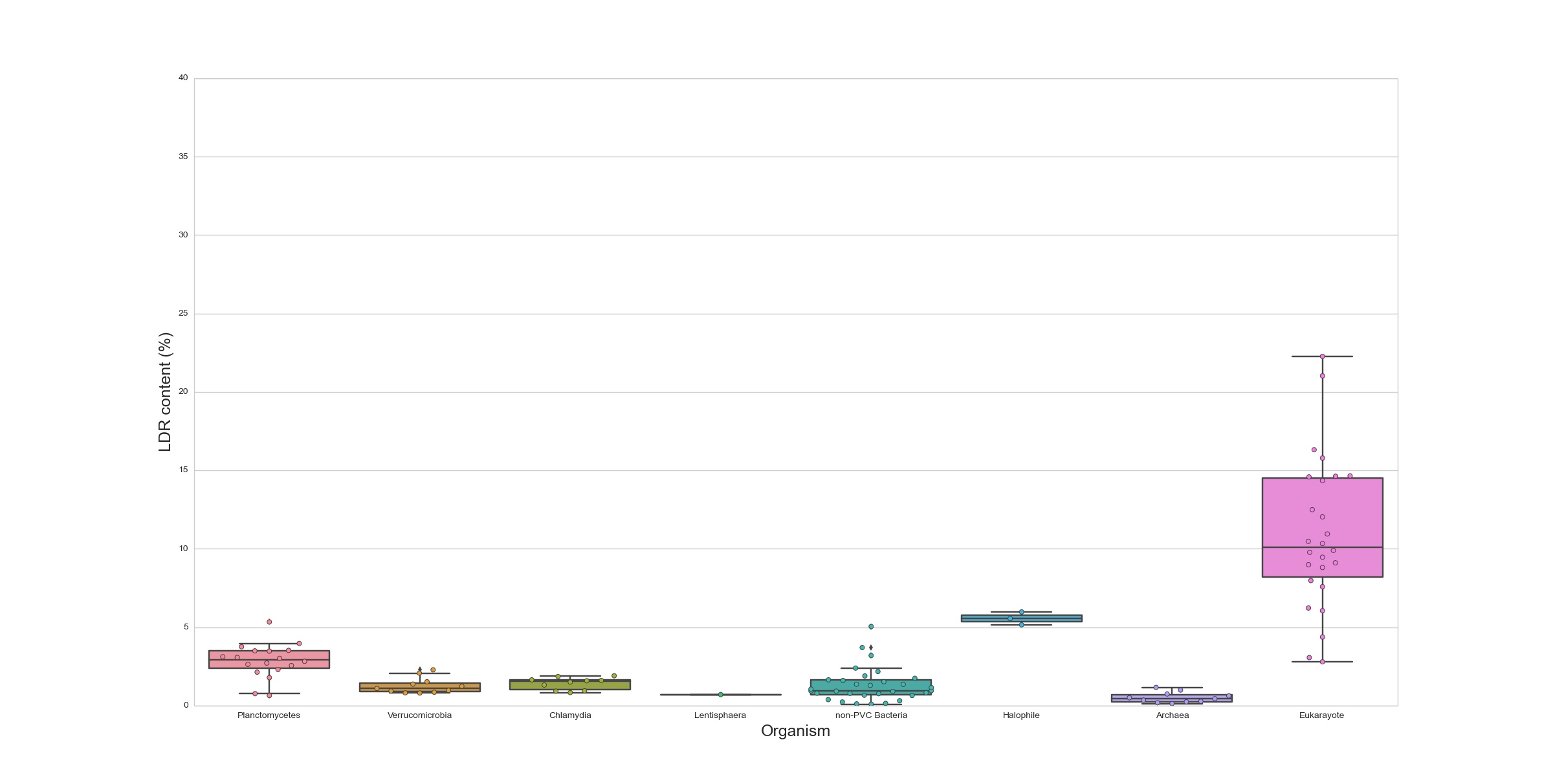

Supplement: Supplementary Data [file bay042_supp.zip › Figure_S2.JPEG]
